# Production and Characterization of Novel Fabs Generated from Different Phage Display Libraries as Probes for Immunoassays for Gluten Detection in Food

**DOI:** 10.3390/foods12173274

**Published:** 2023-08-31

**Authors:** Eduardo Garcia-Calvo, Aina García-García, Santiago Rodríguez, Kristiina Takkinen, Rosario Martín, Teresa García

**Affiliations:** 1 Departamento de Nutrición y Ciencia de los Alimentos, Facultad de Veterinaria, Universidad Complutense de Madrid, 28040 Madrid, Spain; edugar01@ucm.es (E.G.-C.); santro03@ucm.es (S.R.); rmartins@ucm.es (R.M.); tgarcia@ucm.es (T.G.); 2 Biosensors Team, VTT Technical Research Center of Finland Ltd., P.O. Box 1000, FI-02044 Espoo, Finland; kristiina.takkinen@vtt.fi

**Keywords:** gluten, recombinant Fab, celiac disease, phage display, ELISA

## Abstract

Gluten is the main fraction of wheat proteins. It is widely used in the food industry because of the properties that are generated in the dough, but it is also able to trigger diseases like allergies, autoimmunity processes (such as celiac disease), and intolerances in sensitized persons. The most effective therapy for these diseases is the total avoidance of gluten in the diet because it not only prevents damage but also enhances tissue healing. To ensure the absence of gluten in food products labeled as gluten-free, accurate detection systems, like immunoassays, are required. In this work, four recombinant Fab antibody fragments, selected by phage display technology, were produced and tested for specificity and accuracy against gluten in experimental flour mixtures and commercial food products. A high-affinity probe (Fab-C) was identified and characterized. An indirect ELISA test was developed based on Fab-C that complied with the legal detection limits and could be applied in the assessment of gluten-free diets.

## 1. Introduction

Grains are widely recognized as an essential component of a nutritious diet. However, the ingestion of gluten, which accounts for 80–90% of wheat proteins [1], has been linked to several adverse reactions that affect specific and growing population groups. Gluten-related diseases (GRDs) can be classified into three groups based on their etiology: allergic diseases, autoimmune diseases, and a third group that encompasses non-allergic and non-autoimmune gluten intolerance.

In the Western general population, GRDs that involve an allergic component have a prevalence rate of 0.1% [2]. This condition is characterized by a pathogenic immune response mediated by IgE antibodies. The second GRD group corresponds to autoimmune diseases [3]. Celiac disease is the most common of these disorders and is characterized by damage to the lining of the small intestine, which can cause various gastrointestinal and non-gastrointestinal symptoms [4]. The third group corresponds to non-celiac gluten intolerance, a disease that cannot be fit in the previous groups. It has an unknown molecular mechanism, but there is a strong implication of innate immunity [5].

Even though several pharmacological approaches have been developed to mitigate these diseases (especially celiac disease), none has demonstrated a better cost-efficiency than eliminating gluten from the diet [6]. Individuals who are affected by GRD often find it difficult to adhere to a gluten-free diet. Not only do they need to learn which products to avoid, but they also depend heavily on accurate product labeling [7]. The widespread use of gluten in the food industry due to its functional properties adds complexity to this challenge [8]. Without an effective allergen management plan integrated into the Hazard Analysis Critical Control Points (HACCP) system, unintentional contamination of products during production can occur, leading to mislabeling and posing a threat to individuals who are sensitive to gluten.

In the European Union (EU), if a food product contains gluten, it must be clearly labeled as such on the packaging. The labeling must indicate the presence of gluten in the product and include the words “contains gluten” or “contains cereals containing gluten” in the ingredients list or in a separate allergen statement. Additionally, the gluten content of a food product must not exceed 20 mg/kg to be labeled as “gluten-free”, and 100 mg/kg to be labeled as “very low gluten” [9].

To fulfill these requirements, several analytical methodologies have been developed that can be classified into genomic, proteomic, and immunological methods [10]. Genomic methods are based on the detection of the specific genes that allow the identification of gluten-containing species. These genomic methods are widely based on real-time polymerase chain reactions (PCR) [11]. Proteomic methods are based on the detection and quantification of gluten proteins and their peptides by matrix-assisted laser desorption/ionization time-of-flight mass spectrometry (MALDI-TOF MS), which allows reliable determination of protein levels as low as 0.01 mg/mL [12] and can be coupled to HPLC (high-performance liquid chromatography) systems [13] building up a highly reliable methodology. Despite the power of these techniques, they are expensive and applicable only for semi-quantitative measurements [14]. Immunodetection methods are based on antigen–antibody-specific interactions, and they are the most used for gluten detection in food due to their specificity, sensitivity, speed, and accessibility [15]. Among these immunodetection techniques, the application of ELISA (enzyme-linked immunosorbent assay) for gluten detection in food offers a highly sensitive and specific method to quantitatively detect even trace amounts of gluten proteins in food samples. This is essential for regulatory compliance and accurate labeling of gluten-free products. ELISA’s ability to differentiate between potentially harmful gluten components allows for comprehensive analysis, aiding in the identification of potential sources of gluten contamination. By providing a reliable means to assess gluten content, ELISA techniques contribute to safeguarding the health of individuals with gluten sensitivities, enabling them to make informed dietary choices and mitigating the risk of adverse reactions [16].

Immunodetection systems depend on high-affinity antibodies against gluten or its components. The most widely used is the R5 mouse monoclonal antibody [17], and a direct sandwich ELISA based on R5 is considered the gold standard technique for gluten detection in foodstuff [18].

Recombinant antibodies offer numerous advantages over both classical monoclonal and polyclonal antibodies. Firstly, recombinant antibodies are produced using in vitro techniques, avoiding the need for animal immunization, and can be generated in large quantities with consistent batches, ensuring a stable and scalable supply. They also exhibit reduced batch-to-batch variability, increasing the reproducibility and reliability of detection systems [19]. In addition, the recombinant production of antibodies avoids the presence of unwanted light chains generated by the hybridoma cells [20]. Overall, recombinant antibodies stand out as versatile and customizable tools that offer improved performance and sustainability.

Phage display is a vital technique for antibody discovery and engineering. It involves genetically modifying bacteriophages to display antibody fragments on their surface. By fusing genes encoding diverse antibody fragments with the gene codifying a phage coat protein, a library of phages with millions of different displayed antibodies is generated [21]. When exposed to a specific target antigen, phages with high binding affinity for the target are selected. These phages can be isolated, and the corresponding antibody fragments can be further developed into fully functional antibodies. This method enables the efficient identification and isolation of antibodies that have the desired binding characteristics, making phage display a cornerstone in antibody development [22].

**Figure 1:**
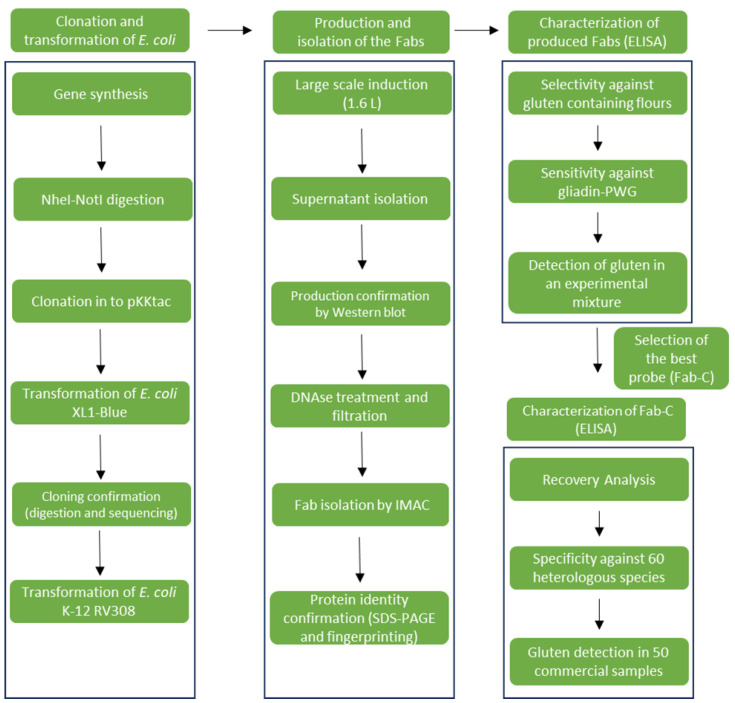
Schematic workflow of the production and characterization of novel Fab as probes in ELISA methods for gluten detection in foodstuff.

Novel single-domain (dAb) recombinant antibodies [23], suitable for the development of gluten detection ELISA systems, have been recently obtained from directed evolution processes based on phage display technology [24]. In this work, the use of Fabs is proposed instead of dAbs because of their enhanced stability and a bigger paratope [25]. The first step for this workflow was building Fab libraries, derived from two strategies: a Fab library merging antibody chains of different origins [26] and an immune library by cloning the genes expressed by peripheral blood lymphocytes from celiac patients [27]. Thanks to the phage display technology, four Fabs were identified from affinity selection processes. The aim of this work was the production of the selected Fabs in a soluble format and studying their feasibility as immunoassay probes for gluten detection in food (Figure 1).

## 2. Materials and Methods

### 2.1. Bacterial Strains and Growth Media

*Escherichia coli* XL1-Blue strain (*rec*A1, *end*A1, *gyr*A96, *thi*-1, hsdR17, *sup*E44, *rel*A1, lac (F, *pro*AB, *lac*IqZΔM15, *Tn*10, (Tetr))) (Agilent©, Santa Clara, CA, USA, ref #200150) was used for subcloning. *E. coli* K-12 strain RV308 substrain ATCC 31608 (*lacI*q, su-, Δ*lacX74*, *gal*, IS II::OP308, *strA*, (DE3)) was used for recombinant Fab production. Luria Broth (LB: 10 g/L tryptone, 5 g/L yeast extract, 10 g/L NaCl, pH 7) was used for growth before the preparation of chemical competent cells. The transformed cells were grown in Super-Broth medium (SB: 30 g/L tryptone, 20 g/L yeast extract, 10 g/L MOPS, pH 7) supplemented with 1% glucose for DNA extraction. The RV308 cells were grown in Terrific Broth medium (TB: 12 g/L tryptone, 24 g/L yeast extract, 4 g/L glycerol, and phosphate buffer (0.17 M KH_2_PO_4_, 0.72 M K_2_HPO_4_, pH 7.2)) for recombinant protein production.

### 2.2. Recombinant Antibodies Selection and Bacterial Transformation

Four gliadin-binding Fab recombinant antibodies (named Fab-C, Fab-H, Fab-E, and Fab8E-4) were previously selected using phage display from Fab libraries cloned into the phagemid pComb3X [26]. The genes coding for the four Fabs were synthesized, changing their original antibody isotype to IgG1 and flanked by restriction sites (*NheI* and *NotI*) for cloning (Eurofins genomics©, Luxemburg, Luxemburg).

The synthetic genes were cloned into the pKKtac expression vector [28] by digestion of the plasmids and inserts with *NheI* and *NotI* restriction enzymes (New England Biolabs© Ipswich, MA, USA ref #R3131 and ref #R3189), purification by gel electrophoresis, and ligation with T4 ligase (Promega©, Madison, WI, USA ref #M1801) overnight at 16 °C.

The resulting ligations were transformed by chemical transformation into *E. coli* XL1-Blue for storage, and into *E. coli* RV308 for protein production.

Chemical-competent cells were in-house produced by growing in 3 mL of SB for 3 h at 37 °C on a shaker (220 rpm). Cells were centrifuged at 4700× *g* for 2 min at room temperature and gently resuspended into 100 µL of ice-cold sterile CaCl_2_ (1 M). The cells were incubated on ice for 30 min. One hundred nanograms of plasmid DNA were added to the chemically competent cells, which were then incubated for 30 min on ice, followed by a 2 min heat shock (37 °C), and incubated again on ice for 2 min. The bacterial cells were recovered by adding 400 µL of SOC medium (Invitrogen™-Thermo Fisher©, Waltham, MA, USA, ref #15544-034) and were grown for 1 h at 37 °C, with shaking at 220 rpm. Then, the culture was plated on LB-ampicillin (100 µg/mL) medium and incubated at 37 °C overnight.

Plasmid DNA was isolated from several transformed *E. coli* RV308 colonies using NucleoSpin^®^ Mini kit for plasmid DNA (Machery-Nagel©, Allentown, PA, USA, ref # 740588.50). Plasmids were digested with *NheI* and *NotI* and analyzed by agarose gel electrophoresis (Thermo-fisher©, ref #16520050) and by Sanger sequencing to ensure that the clonation process was correct.

### 2.3. Production of Recombinant Antibodies

A single colony of *E. coli* RV308, transformed with the plasmid codifying the Fab of interest, was inoculated into 50 mL of TB medium supplemented with 1% glucose and 100 µg/mL ampicillin and incubated overnight at 37 °C with shaking at 220 rpm. Then, this culture was expanded to 1.8 L of TB medium supplemented with 100 µg/mL ampicillin, and divided into 6 flasks that were shaken (220 rpm) at 37 °C until the OD_600_ reached 4. The recombinant production was induced using 1 mM of IPTG (Isopropyl β-D-1-thiogalactopyranoside), lowering the temperature to 30 °C, and shaking at 170 rpm overnight. Finally, the recombinant Fabs were recovered from the overnight culture by centrifugation at 4700× *g* for 15 min.

Production of the recombinant Fabs was assessed by Western blot analysis. Supernatant proteins were separated by electrophoresis in a 15% polyacrylamide gel at 100 V, using Biorad dual color as the molecular weight marker (BioRad, Hercules, CA, USA ref #1610364). The separated proteins were transferred to a PVDF (Polyvinylidene Difluoride) membrane at 100 V for 1 h. The PVDF membrane was blocked with TBST (50 mM Tris HCl, 150 mM NaCl, Tween20 0.05%, pH 7.5) for 30 min at 37 °C. Goat anti-human IgG F(ab)_2_ alkaline phosphatase conjugate (Rockland©, Philadelphia, PA, USA ref #709-1518) diluted 1:1000 in TBST was added and incubated for 1 h at room temperature. The membrane was rinsed 3 times with TBST before the addition of 5-bromo-4-chloro-3-indolyl-phosphate and Nitroblue-Tetrazolium (BCIP/NBT) to visualize the bands containing Fab.

The supernatants containing the Fabs were treated with DNAse I (Merck© Darmstadt, Germany ref #11284932001) and filtered through Whatman^®^ glass filters GF/B and GF/C (Merck© ref #182110 and ref #182202) in a suction bottle to reduce viscosity. Then, the supernatant was nine-fold concentrated, and the buffer was exchanged for PBS-imidazole (137 mM NaCl, 2.7 KCl, 10 mM Na_2_HPO_4_, 1.8 mM KH_2_PO_4_, 5 mM imidazole, pH 7.4) with a prep/scale tangential flow filtration (TFF) 1 ft^2^ cartridge (Merck©, ref #CDUF001LG). The concentrated supernatant was centrifuged at 15,000× *g* for 10 min at room temperature and filtered through Whatman^®^ glass filters GF/F and GF/D (Merck© ref #1825090 and ref #182390) in a suction bottle.

### 2.4. IMAC-Purification and Analysis

The recombinant Fabs were purified using the NGC discovery chromatography system (BioRad, ref #7880009) and a HisTrap FF crude 5 mL affinity column (Cytiva, Marlborough, MA, USA, ref #11-0004-58) charged with Nickel ions from a NiSO_4_ 100 mM solution. Two buffers were used for the purification: a binding buffer (20 mM NaH_2_PO_4_, 500 mM NaCl, 5 mM imidazole, pH 7.4) and an elution buffer (20 mM NaH_2_PO_4_, 500 mM NaCl, 500 mM imidazole, pH 7.4).

Once the column was equilibrated, the treated supernatant was fed to the system with a 5 mL/min flow rate. Then, the unbound proteins were flushed away by a washing program (5 mL/min flow rate and 5 column volumes of binding buffer). The proteins bound to the column were eluted by a gradient of elution (2 mL/min flow rate from 5 to 500 mM of imidazole in 75 min). A final wash with elution buffer was done to strip off every remaining protein.

The process was followed by spectrophotometry, measuring the output flow at 280 nm, and samples from each fraction of interest were analyzed by SDS-PAGE (sodium dodecyl-sulfate polyacrylamide gel electrophoresis). The fractions that contained the recombinant Fabs were dialyzed against 100 volumes of PBS (137 mM NaCl, 2.7 KCl, 10 mM Na_2_HPO_4_, 1.8 mM KH_2_PO_4_, pH 7.4) overnight at 4 °C, for imidazole elimination.

To confirm the presence of Fabs, the bands from the SDS-PAGE, which matched with the Fab size, were analyzed by mass fingerprint. The corresponding bands were excised using a sterile scalpel. Following the methodology described by Sechi and Chait [29], the samples underwent in-gel reduction, alkylation, and trypsin digestion. After overnight trypsinization, 1 µL of the resulting supernatant was allowed to dry and then spotted onto a MALDI plate. The plate was prepared using α-cyano-4-hydroxy-cinnamic acid matrix (Sigma) in 50% acetonitrile. Subsequently, peptide analysis was conducted at the Proteomics Unit of Complutense University of Madrid (Spain) using a 4800 Plus Proteomics Analyzer MALDI-TOF/TOF instrument (Applied Biosystems, MDS Sciex, Toronto, ON, Canada). Protein identification via peptide mass fingerprinting (PMF) was performed using the MASCOT v2.6.2 search engine through Global Protein Server (GPS) v.3.6 (ABSCIEX, Toronto, ON, Canada), employing the following search parameters: carbamidomethylcysteine as a fixed modification, oxidized methionine as a variable modification, allowing one missed trypsin cleavage site, and with peptide mass tolerance set to 80 mg/kg and MS/MS fragment tolerance to 0.3 Da.

### 2.5. Reference Materials and Food Samples Used for Analysis

Gliadin-PWG (prolamin working group) was used as reference material. Gliadin-PWG was obtained from the ethanolic extraction of a mixture of 28 European wheat cultivars after the elimination of albumins and globulins using a 0.4 M NaCl solution and then were concentrated, desalted by ultrafiltration, freeze-dried, and homogenized [30].

To test the ability of soluble Fabs obtained to detect gluten in complex matrixes, an experimental mixture of gluten-free rice flour spiked with flour made of kernels of gluten-containing cereals was prepared, as previously described [31]. The kernels, kindly provided by the national seed repository (Instituto Nacional de Investigaciones Agrarias, INIA, Spain), were ten different cultivars of each of the following cereals: common wheat (*Triticum aestivum vulgare*), spelt wheat (*Triticum aestivum spelta*), rivet wheat (*Triticum turgidum turgidum*), durum wheat (*Triticum turgidum durum*), barley (*Hordeum vulgare*), rye (*Secale cereale*), and two cultivars of the hybrid crop triticale (×*Triticosecale*). All the mixtures were ground and mixed in an IKA A11 analytical mill (IKA^®^, Staufen, Germany).

To test specificity for gluten, 60 heterologous species not containing gluten were tested to ensure there was no cross-reactivity (Table 1).

In addition, 50 commercial food products were purchased from several local stores (Spain). According to their labels, they were classified into 9 gluten-containing products, 6 not declaring gluten, 21 declaring that they could contain gluten, and 14 labeled as gluten-free.

### 2.6. Extraction of Gluten from Samples

Firstly, 50 g of each tested sample was individually ground in the analytical mill, ensuring thorough cleaning between samples, and stored at −20 °C. The gluten-like proteins were extracted from 250 mg of finely ground samples (either experimental mixtures, spiked samples, or food products) using 2.5 mL of the Ingezim Gluten extraction solution (Ingenasa©, Madrid, Spain). The mixture was homogenized, incubated at 50 °C for 40 min, and cooled to room temperature before addition of 7.5 mL of 80% ethanol/water solution and shaking for 1 h in a vertical rotator. The extract was centrifuged at 2000× *g* for 10 min at room temperature, and the supernatant containing gluten proteins was transferred to a glass vial and stored in darkness at 23 °C until used.

### 2.7. Indirect Enzyme-Linked Immunosorbent Assay (ELISA) Method Based on the Recombinant Fabs

One hundred microliters of the ethanolic sample extracts obtained as described above (diluted 1/5 in PBS), or a solution of the appropriate gliadin-PWG concentration, was added per well. The immune-sorbent plate (Thermo©, ref #163320) was sealed and incubated for 1 h at 37 °C. The coating solution was shaken out, 200 µL blocking solution (3% BSA in PBS) was added per well, and the plate was incubated for 1 h at room temperature. Following 10 washing steps with PBS, 0.5 µg of recombinant Fab was added per well, diluted in 100 µL of blocking solution, and incubated for 1 h at room temperature in a plate shaker. The plate was washed 10 times with PBS, and the secondary antibody (rabbit anti-human H + L HRP conjugated, Abcam©, Cambridge, UK ref #6759), diluted in 100 µL of blocking solution was added, and incubated for 1 h at room temperature in a plate shaker. After washing 10 times with PBS, 100 µL of TMB (Sigma©, ref #T0440) was added. The reaction was stopped after 20 min with 50 µL of a diluted sulphuric acid solution, and the signal detected at 450 nm.

### 2.8. Assay Validation

The mean values of three independent determinations and standard derivation of each data set are shown in the figures. The data were plotted, and fitting models were determined using Origin 8.0 software (OriginLab Corp., Wellesley Hills, MA, USA). The limits of detection (LOD) and quantification (LOQ) were calculated as three and ten times the standard deviation of ten blank replicates, respectively [32].

The recovery of gliadin from food samples was analyzed by using a gluten-free certified rice flour spiked with solid gliadin-PWG equivalent to theoretical 20 and 100 mg/kg of gluten. As a control, a rice-flour ethanol extract was spiked with the equivalent quantities of ethanol-dissolved gliadin-PWG (0.05 µg/mL and 0.25 µg/mL).

ELISA results obtained from 50 commercial food products were compared with those obtained with R5 monoclonal antibody-based sandwich ELISA (Ingenasa©, ref #30.GL2.K.2), which has a LOQ of 3 mg/kg of gluten and has been approved as a Type I method by *Codex Alimentarius*, following the manufacturer protocol.

## 3. Results and Discussion

The phage display technology has witnessed several applications concerning gluten, mainly in clinical contexts but also for its detection in food. Immunized llamas [33] and a semi-synthetic library [34] have been used for these purposes. However, this study marks the inaugural production and characterization of antibody fragments derived from immune libraries sourced from celiac patients’ peripheral blood. These fragments, whether originating from total or partial immune libraries, have been deployed for gluten detection in food.

### 3.1. Expression and Purification of Recombinant Fabs

Four gliadin-binding recombinant phage-Fabs were selected by phage-display for their soluble expression and characterization from two libraries of different origins previously obtained by our group. One of the Fabs (Fab8E-4) was isolated from a library that resulted from merging semi-synthetic heavy chains and immune light chains [26], and the other three (Fab-C, Fab-E, and Fab-H) were isolated from a fully immune phage-display library [27].

To enhance the recombinant expression of Fabs, the original isotypes found after their isolation from the library were switched to IgG1. The isotype change allowed the use of the same secondary antibody for different primary Fabs, increasing test uniformity. It should be noticed that most of the antibodies selected from the immune library presented an IgA isotype, an antibody class that presents an extra disulfide bond, which challenges the production and folding of the recombinant protein [35]. However, it was not surprising to find IgA antibodies because the library they were isolated from was produced from peripheral blood lymphocytes from celiac disease patients, and celiac disease is a mucosal autoimmune condition where gluten-directed IgA antibodies are generated [36].

**Figure 2:**
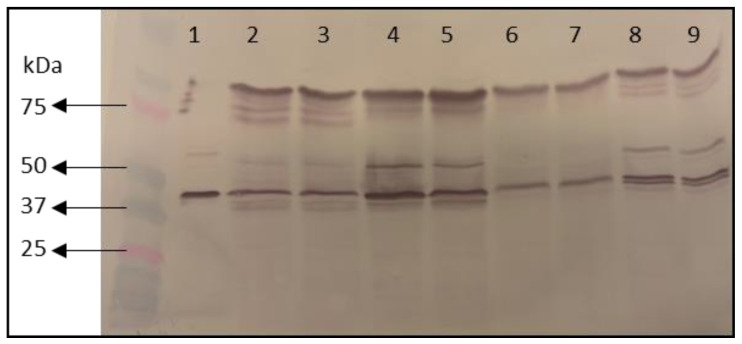
Western blot analysis of transformed *E. coli* RV308 culture supernatants after induction for expression of recombinant Fabs. Lane 1: control Fab previously produced; Lanes 2 and 3: Fab-C; Lanes 4 and 5: Fab-E; Lanes 6 and 7: Fab-H; Lanes 8 and 9: Fab8E-4.

The recombinant Fabs were expressed in the supernatant thanks to the combination of the clonation of the antibody chains after a *pelB* secretion signal and the weak cell wall of the bacterial strain used. After growing and inducing the transformed colonies for recombinant production, a Western blot was performed to confirm the effective expression of the Fabs in the supernatant prior to purification. All the supernatants contained bands coincident with the Fab size (approx 50 kDa), and the size of the Fab used as a control. Apart from the Fab, other bands in the gel are proteins secreted to the medium by *E. coli*. These results demonstrated the suitability of the strategy used for the heterologous expression of the selected Fabs and allowed the progression of the purification process (Figure 2).

**Figure 3:**
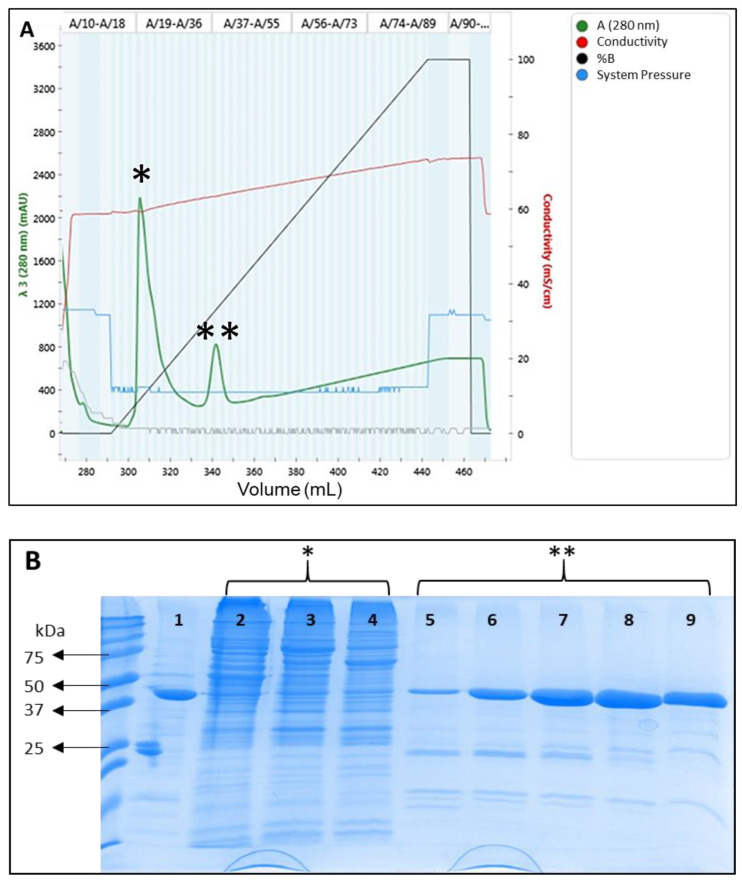
Recombinant production, purification, and identification of Fabs. (**A**) Example of the immobilized metal affinity chromatography (IMAC) profile for the purification of the recombinant Fabs (Fab-C in this image). In green, monitorization of the protein quantity (absorbance 280 nm) going through the column. In red, conductivity measures. In black, percentage of elution buffer pumped to the system. In blue, measure of the system pressure. (**B**) Analysis of the purification steps by SDS-PAGE electrophoresis. Lane 1: control Fab previously produced. Lanes 2–4: samples from different fractions representing the first peak of the chromatogram (*). Lanes 5–9: samples from different fractions representing the second peak of the chromatogram (**) containing the desired Fab.

The antibody fragments, produced as histidine-tagged proteins, were purified from the cleared supernatant through IMAC (Immobilized Metal Affinity Chromatography). The isolation process was monitored by spectrometry (Figure 3A). When the imidazole gradient was activated, the bound proteins were eluted, producing two significant protein signal peaks that were analyzed by SDS-PAGE (Figure 3B). The proteins eluted in the first peak were *E. coli* proteins bound to the column and those from the second peak, the Fabs. The isolation of the target Fabs was confirmed by mass fingerprinting of the band with the appropriate molecular weight (approx 50 kDa) of a Fab. For Fab-C, the hit analysis of the mass fingerprint against the sequence of the Fab showed a protein score (a measure of the probability that the observed match is a random event) of 84, when the statistical analysis indicated that scores greater than 13 are significant (*p* < 0.05). Among the tryptic peptides identified, there were some containing part of the constant regions and the HCDR3 (third Complementarity-Determining Region of the Heavy Chain) that allowed a clear identification of the Fab-C.

This data confirmed that the Fabs were found in fractions eluted at a concentration of approximately 100 mM of imidazole.

To ensure that the desired antibody fragment was obtained with an acceptable level of purity and homogeneity, the purified fraction was dialyzed to remove the imidazole. The recombinant production strategy resulted in the obtention of between 6 and 9 milligrams per antibody, a high yield for *E. coli* production using non-continuous cultures (flasks) [37]. Moreover, the proposed methodology has been proven as a fast, accurate, and reproducible option to produce recombinant Fabs, allowing high uniformity between batches of the same and also different antibodies [38].

### 3.2. Indirect ELISA Development and Validation for Gluten Detection

**Figure 4:**
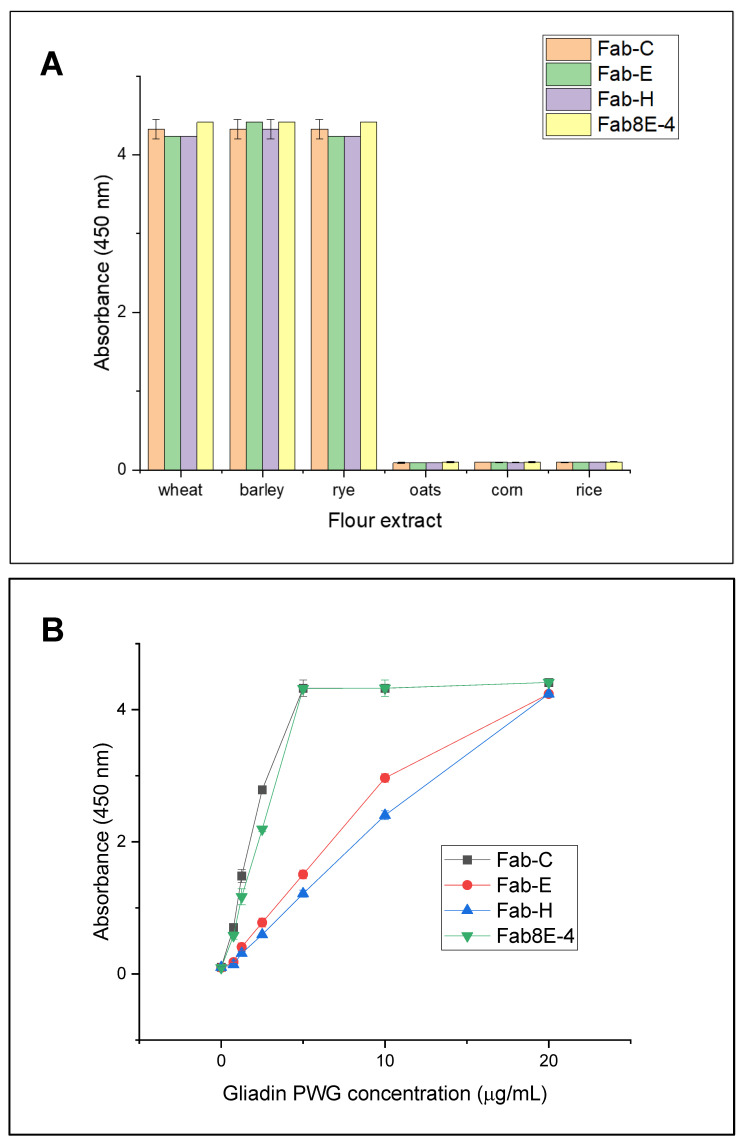

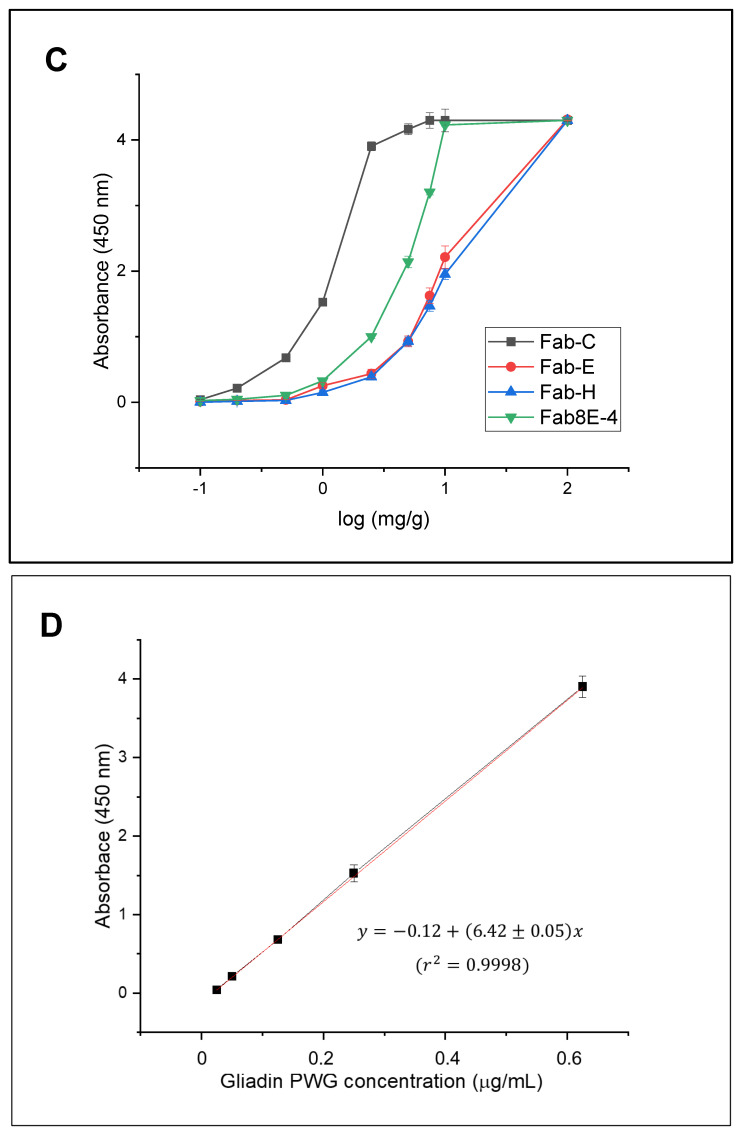
Indirect ELISA results for characterization of the four recombinant Fabs produced. (**A**) Specificity results against ethanolic extracts of gluten-containing (wheat, barley, and rye) and gluten-free (oats, corn, and rye) flours (diluted 1:5 in PBS). (**B**) Sensitivity evaluation by comparative dose-response curves obtained against gliadin-PWG (0–20 µg/mL) diluted in PBS. (**C**) Comparison of dose–response curves for detection of gluten in an experimental mixture (rice flour spiked with growing concentrations of wheat, rye, and barley flours). (**D**) Linear regression results obtained from the analysis of the gliadin-PWG standard (0.025–0.625 µg/mL) by indirect ELISA using Fab-C as primary antibody.

The specificity of the recombinant Fabs for gluten was thoroughly evaluated by an indirect ELISA, using the purified recombinant Fabs as probes and analyzing immunoplates coated with wheat, barley, and rye as the target species and maize, oats, and rice flours as non-target species (Figure 4A). As expected, the soluble Fabs antibodies did not exhibit any cross-reactivity with the cereals not containing gluten despite their close phylogenetic relationship.

Once the specificity of the four Fabs was demonstrated, the sensitivity of the indirect ELISA using the different purified recombinant Fabs was evaluated by analyzing gliadin-PWG reference material (Figure 4B). The results showed that Fab-C exhibited the highest sensitivity, followed by Fab8E-4. Conversely, Fab-E and Fab-H demonstrated comparatively lower sensitivity.

The performance of the indirect ELISA test with soluble Fabs was not only evaluated against purified gliadin but also in a matrix that resembles the intended application for gluten detection in food. To this end, an ELISA test was performed to detect gluten in an experimental flour mixture consisting of increasing concentrations of gluten containing wheat, barley, and rye flours blended with a rice flour matrix (non-containing gluten) (Figure 4C). These results reinforced those obtained from the gliadin-PWG curves, and the Fabs demonstrated consistent performance in different tests.

A gluten recovery analysis was performed by testing the gluten-like proteins extracted from a gluten-free certified rice flour spiked with solid gliadin-PWG (equivalent to theoretical 20 and 100 mg/kg of gluten) with the indirect Fab-C ELISA. The actual gluten content of these mixtures was assessed by analysis with the R5 sandwich ELISA. The results of the recovery test for the methodology proposed were 76.71 ± 1.72% for the 20 mg/kg mixture and 90.89 ± 0.36% for the 100 mg/kg mixture. As a control, a rice flour extract was spiked with the equivalent quantities of dissolved gliadin-PWG (0.05 μg/mL, equivalent to 20 mg/kg in food samples, and 0.25 μg/mL, equivalent to 100 mg/kg). The results for these controls were 79.93 ± 3.35% for the 0.05 μg/mL solution and 92.11 ± 1.07% for the 0.25 μg/mL solution. All the results obtained presented an appropriate recovery limit in the 75–125% range [39].

In summary, the results demonstrated that Fab-C exhibited the strongest response against gluten, using purified gliadin-PWG and gluten-like proteins extracted from an experimental flour mixture.

Moreover, 60 heterologous samples were analyzed by an indirect ELISA to assess any potential cross-reactivity. The results demonstrated that Fab-C exhibited no cross-reactivity with these matrices, indicating its high specificity for gluten detection. These findings suggest that Fab-C may be a reliable and accurate tool for gluten immunodetection in food products, as it can effectively differentiate between gluten-containing and non-gluten-containing samples.

The sensitivity of the Fab-C-based indirect ELISA was evaluated using a linear model based on increasing concentrations of gliadin-PWG, ranging from 0.025 to 0.625 µg/mL (Figure 4D). The limit of detection (LOD) was calculated as 0.028 µg/mL by interpolating three times the standard deviation of ten blanks. This corresponds to a gluten concentration of 11 mg/kg in the samples (considering a dilution factor of 5, an extraction factor of 40 (0.25 g of sample extracted with 10 mL of buffer), and the conversion factor of gliadin to gluten, 2). The limit of quantification (LOQ) was determined by interpolating ten times the standard deviation of the blank, being 0.05 µg/mL of gliadin-PWG, equivalent to 19.8 mg/kg of gluten. These results confirm that the indirect ELISA using Fab-C recombinant antibody fulfills the limits established by the current legislation, and it will differentiate gluten-free products (less than 20 mg/kg of gluten) from gluten-containing ones.

The sensitivity of the assay is slightly lower than that of the R5 sandwich ELISA, which could be explained by the different types of assay and antibodies involved. The developed indirect ELISA shows a single molecular interaction per antigen molecule because the Fab possesses only one paratope, thereby allowing for a single binding event per Fab. Conversely, the R5 method involves two molecular interactions with the antigen due to the presence of two paratopes per whole antibody molecule and the utilization of a direct sandwich format, enabling dual interactions involving the capture and detection of antibodies [40]. Considering these factors, it is demonstrated that the single-paratope molecule (Fab-C) exhibits a high affinity towards the target antigen.

### 3.3. Detection of Gluten in Commercial Food Products by Indirect ELISA Based on the Recombinant Fab-C

Once the proposed indirect ELISA with Fab-C demonstrated good specificity, sensitivity, and gluten recovery features, the methodology was applied to analyze gluten-like proteins extracted from a wide variety of commercial food products, including cereal, dairy, meat, and drinks, processed and raw products.

Samples were sorted according to their labeling in four different categories: (A) products that declared to contain gluten (9 samples); (B) products with “may contain gluten” precautionary labeling (6 samples); (C) products that did not declare gluten or that did not specifically warn of the presence of gluten (21 samples); and (D) products with “gluten-free” labeling and/or certification (14 samples).

To assure the accuracy of the proposed test, the food samples were also analyzed by the gold standard technique for gluten detection in food, a direct sandwich ELISA based on the monoclonal antibody R5. The results are summarized in Table 2. Most of the samples analyzed produced the same result with both methods and showed good agreement with labeling regarding the classification of the food samples following the guidelines of European legislation.

From the 50 products analyzed with the Fab-C indirect ELISA, the results of 47 matched with the validation technique used (R5-ELISA). However, three products tested positive by the R5 reference assay but negative by the Fab-C indirect ELISA. It is remarkable that the samples that did not conform with the R5 standard test contained oats as the main ingredient. It is hypothesized that this result can be attributed to the absence of cross-reactivity exhibited by Fab-C with oats. It has been demonstrated that certain oat cultivars produce cross-reactivity when analyzed with other detection antibodies like R5, reporting up to 100 mg/kg of gluten in some oats cultivars [41]. The cross-reaction of the R5 monoclonal antibody has been explained due to the high similarity of certain peptides found in avenins (gluten-like proteins in oats) with gluten peptides [42]. The absence of this unintended detection in gluten-free oat-derived products could mean a stronghold of Fab-C, and it could help in the assessment of gluten in these types of products, especially those containing cultivars that are cross-reactive with other antibodies in the market. In light of these results, the proposed methodology demonstrated a reliable capacity for discriminating gluten-free products in the samples analyzed.

Detection of gluten in foodstuff is still a major challenge in food science because the concept of gluten is defined by chemical features (ethanol solubility), encompassing a very big range of proteins (unlike other food allergens that are represented by one or few proteins) that present some common characteristics but can differ in their sequences [43]. This fact makes it very hard to discover a universal probe for gluten detection. Several comparative studies unveiled that the commercial kits available in the market presented important discrepancies between them for the quantification of gluten [40,44]. These differences were explained by the different epitopes of gluten that the antibodies were binding to. For example, the R5 antibody recognized epitopes that were more expressed in rye, barley, or triticale than in wheat, in contrast to the G12 monoclonal antibody, which presented a better detection of wheat epitopes [45]. The recognition of wheat, rye, and barley was very similar in an indirect ELISA with the recombinant Fabs produced in this work (Figure 4A). Moreover, although R5 and G12 monoclonal antibodies each detected different epitopes, all were found in the N-terminal zone of the gliadins. On the contrary, computational studies with some of the antibodies developed in this work presented interactions with the gliadin C-terminal portions like Fab8E-4 [26] and Fab-C [27]. The methodology applied in this work for generating Fabs (construction of celiac-derived Fab libraries for phage display selection) allowed the transference of some features from the humoral response of celiac patients to the obtained probes instead of the classical approach of using polyclonal or monoclonal antibodies, generated by a forced immune response of experimentation animals [13]. This feature could be important, as the recombinant probes produced are prone to recognize those epitopes that may be harmful to gluten-sensitive patients.

## 4. Conclusions

Using the pKKtac plasmid and *E. coli* RV308 expression system, four soluble recombinant Fabs obtained from two different phage display libraries (immune and merged semi-synthetic) were produced. This recombinant production methodology provides a cost-effective and efficient alternative to traditional antibody production methods, which can be time-consuming and expensive. Moreover, recombinant antibodies also offer a more consistent and reproducible approach to antibody production, allowing for the availability of standardized affinity probes. All these benefits are achieved without the need to use animals.

The four recombinant Fabs produced selective reactivity to gluten, but Fab-C demonstrated the best detection features in indirect ELISA methodology. Fab-C was capable of detecting gliadin traces at very low concentrations, as low as 28 ng/mL equivalent to 11 mg/kg in food samples, not showing cross-reactions with all the analyzed heterologous species that do not contain gluten. In addition, the ELISA test developed meets the legislative requirements for identifying gluten-free products, and it could have a desirable feature not covered by some commercial tests, as it does not cross-react with oat proteins.

## Figures and Tables

**Table 1:** List of heterologous species (common name and scientific name) that did not show cross-reactivity when tested in the indirect ELISA with Fab-C.

Okra (*Abelmoschus esculentus*)	Lentil (*Lens culinaris*)
Button mushroom (*Agaricus bisporus*)	Cassava (*Manihot esculenta*)
Onion (*Allium cepa*)	Mango (*Mangifera indica*)
Leek (*Allium ampeloprasum*)	Red banana (*Musa acuminata*)
Cashew (*Anacardium occidentale*)	Myrtle (*Myrtus communis*)
Peanut (*Arachis hypogaea*)	Olive (*Olea europaea*)
Strawberry tree (*Arbutus unedo*)	Rice (*Oryza sativa*)
Beetroot (*Beta vulgaris*)	Passion fruit (*Passiflora edulis*)
Cabbage (*Brassica oleracea*)	Pepperomia (*Peperomia pellucida*)
Chinese cabbage (*Brassica rapa subsp. pekinensis*)	Avocado (*Persea americana*)
Pigeon pea (*Cajanus cajan*)	Common bean (*Phaseolus vulgaris*)
Chili pepper (*Capsicum annuum*)	Runner bean (*Phaseolus coccineus*)
Tabasco pepper (*Capsicum frutescens*)	Cape gooseberry (*Physalis peruviana*)
Scotch bonnet pepper (*Capsicum chinense*)	Pea (*Pisum sativum*)
Papaya (*Carica papaya*)	Almond (*Prunus dulcis*)
Chickpea (*Cicer arietinum*)	Sweet almond (*Prunus dulcis var. dulcis*)
Lemon (*Citrus limon*)	Plum (*Prunus domestica*)
Mandarin orange (*Citrus reticulata*)	Raspberry (*Rubus idaeus*)
Watermelon (*Citrullus lanatus*)	Atlantic salmon (*Salmo salar*)
Cantaloupe (*Cucumis melo var. cantalupensis*)	Castor bean (*Ricinus communis*)
Cucumber (*Cucumis sativus*)	Blackberry (*Rubus fruticosus*)
Quince (*Cydonia oblonga*)	Sesame (*Sesamum indicum*)
Persimmon (*Diospyros kaki*)	Tomato (*Solanum lycopersicum*)
Teff (*Eragrostis tef*)	Cherry tomato (*S. lycopersicum cerasiforme*)
Arugula (*Eruca vesicaria subsp. sativa*)	Spinach (*Spinacia oleracea*)
Strawberry (*Fragaria x ananassa*)	Vanilla (*Vanilla planifolia*)
European strawberry (*Fragaria vesca*)	Adzuki bean (*Vigna angularis*)
Soybean (*Glycine max*)	Mung bean (*Vigna radiata*)
Sunflower (*Helianthus annuus*)	Grape (*Vitis vinifera*)
Walnut (*Juglans regia*)	Ginger (*Zingiber officinale*)

**Table 2:** Results obtained for the detection of gluten in commercial food products using the developed Fab-C-based indirect ELISA and the sandwich monoclonal R5 antibody for result confirmation.

Products	No. of Products	Indirect Fab-C	Sandwich R5
(A) Products declared to contain gluten in the labeling (9)			
Pasta	2	+(2)	+(2)
Soup or rice plates	3	+(3)	+(3)
Oats flakes	1	+(1)	+(1)
Cereal bars	2	+(2)	+(2)
Dairy products	1	+(1)	+(1)
(B) Products with “may contain” precautionary labeling (6)			
Cereal	4	+(3)/−(1)	+(3)/−(1)
Flakes	2	−(2)	−(2)
(C) Products that did not declare gluten or that did not specifically warn of the presence of gluten (21)			
Cereal products	9	+(7)/−(2)	+(7)/−(2)
Oat drinks	3	+(1)/−(2)	+(1)/−(2)
Oat flakes	8	+(2)/−(6)	+(5)/−(3)
Meat	1	−(1)	−(1)
(D) Products with “gluten free” labeling and/or certification (14)			
Cookies/cakes	6	−(6)	−(6)
Pasta	1	−(1)	−(1)
Baby food	2	−(2)	−(2)
Corn flakes	1	−(1)	−(1)
Oat flakes	1	−(1)	−(1)
Meat products	3	−(3)	−(3)

Minus sign (−) indicates gluten content lower than the legal limit of 20 mg/kg of gluten, which allows the labeling with “gluten free” statement, and plus sign (+) indicates values above the mentioned legal limit. The gliadin PWG standard curve was used as reference for the indirect Fab-C ELISA and the R5 Sandwich ELISA.

## Data Availability

Data is contained within the article.
